# Supplementary material for: GTP-dependent formation of straight tubulin oligomers leads to microtubule nucleation
Source: J Cell Biol. 2021 Feb 5;220(4):e202007033. doi: 10.1083/jcb.202007033 (PMC7871348; doi:10.1083/jcb.202007033)
Supplement: Table S1 — shows data collection and refinement statistics. [file JCB_202007033_TableS1.docx]

**Table S1.** Data collection and refinement statistics

|  | WT(GDP) | WT(GMPCPP) | Y222F(GDP) | Y222F(GTP) |
| --- | --- | --- | --- | --- |
| **Data collection*** |  |  |  |  |
| Space group | P2_1_2_1_2_1_ | P2_1_2_1_2_1_ | P2_1_2_1_2_1_ | P2_1_2_1_2_1_ |
| Cell dimensions |  |  |  |  |
| *a*, *b*, *c* (Å) | 66.6 126.5 250.2 | 66.7 126.6 251.0 | 66.2 126.7 250.4 | 66.0 126.3 249.8 |
| Resolution (Å) | 48 - 2.30  (2.34-2.30) | 48.2-2.29  (2.33-2.29) | 63.4 – 2.20  (2.23-2.20) | 50.0 – 3.57  (3.66-3.57) |
| R_meas_ | 0.092 (1.13) | 0.112(1.87) | 0.125 (1.25) | 0.51 (3.54) |
| *I*/σ*I* | 10.3 (1.0) | 13.2 (1.2 | 12.8 (1.6) | 4.1 (0.7) |
| CC 1/2 | 0.999 (0.611) | 0.999 (0.612) | 0.998 (0.912) | 0.979 (0.41) |
| Completeness (%) | 98.8 (92.1) | 99.5 (89.8) | 99.5 (91.2) | 98.7 (83.1) |
| Multiplicity | 4.1 (3.4) | 7.4 (6.6) | 9.4 (6.2) | 11.7 (8.9) |
|  |  |  |  |  |
| **Refinement** |  |  |  |  |
| Resolution (Å) | 2.30 | 2.29 | 2.20 | 3.57 |
| No. reflections | 87,343 | 91,616 | 104,968 | 25301 |
| *R*_work/_ *R*_free_ | 0.183/0.218 | 0.187/0.217 | 0.167/0.208 | 0.221/0.244 |
| No. atoms |  |  |  |  |
| Protein | 14509 | 14560 | 14453 | 14483 |
| Ligand/ion | 189 | 193 | 199 | 185 |
| Water | 550 | 432 | 759 | 0 |
| B-factors |  |  |  |  |
| Protein | 68.1 | 64.2 | 52.6 | 111 |
| Ligand/ion | 75.6 | 75.0 | 68.6 | 88.7 |
| Water | 63.7 | 60.1 | 54.0 |  |
| R.m.s deviations |  |  |  |  |
| Bond lengths (Å) | 0.008 | 0.008 | 0.010 | 0.008 |
| Bond angles (º) | 0.93 | 0.98 | 1.08 | 0.98 |
| Coordinate error (Å)  Ramachandran (%)  Favored region  Allowed region  Outliers  **PDB Code** | 0.33  97.48  2.02  0.49  6TIS | 0.32  97.50  2.12  0.38  6TIY | 0.26  96.87  2.91  0.22  6TIZ | 0.60  96.77  2.96  0.27  6TIU |

*Data were collected on a single crystal. Highest resolution shell is shown in parenthesis.
